# Noninvasive positive pressure ventilation for treating acute asthmatic attacks in three pregnant women with dyspnea and hypoxemia

**DOI:** 10.1002/ccr3.2117

**Published:** 2019-03-22

**Authors:** Hiroshi Sekiguchi, Yutaka Kondo, Tatsuma Fukuda, Kazuhiko Hanashiro, Motoo Baba, Yoko Sato, Ichiro Kukita, Tuyoshi Matumoto

**Affiliations:** ^1^ Pulmonary Medicine Tomishiro Central Hospital Tomigusuku Japan; ^2^ Department of Emergency and Critical Care Medicine, Graduate School of Medicine University of the Ryukyus Nishihara Japan; ^3^ Department of Emergency Medicine Juntendo University Urayasu Hospital Chiba Japan; ^4^ Department of Public Health and Hygiene, Graduate School of Medicine University of the Ryukyus Nishihara Japan; ^5^ Pulmonary Medicine Ohama Daiichi Hospital Naha Japan

**Keywords:** acute asthma exacerbation, dyspnea, noninvasive positive pressure ventilation, oxygen therapy, pregnancy

## Abstract

In our case reports, we mentioned about the utility of NPPV therapy in addition to standard pharmacologic therapy for acute asthma exacerbations in pregnant women with dyspnea and hypoxemia compared with that of oxygen therapy alone. Careful patient selection and clinicians’ NPPV experience are crucial in optimizing patient outcomes.

## 1 INTRODUCTION

According to the World Health Organization, an estimated 235 million people suffer from asthma. Despite the elucidation of the pathophysiology of asthma, advancements in inhalation therapies (bronchodilator and steroid), and promotion of educational programs for preventing asthma attacks, there were 383 000 asthma‐related deaths in 2015.^1^ Bolz et al^2^ have reported that asthma is the most common chronic lung disease in pregnant females, with a prevalence of 4%‐8%. Murphy et al^3^ have reported that asthma affects 3%‐12% of pregnant women worldwide. Therefore, effective management and prevention of acute asthma exacerbations during pregnancy are important to maintain the health of both the mother and fetus.^3^ Medical prophylaxis of asthma attacks is fundamental for asthma treatment. When an asthma attack occurs in the primary‐care setting, the patient must be transferred to an acute‐care facility to undergo controlled oxygen therapy, inhalation therapy with short‐acting beta‐2 agonists and ipratropium bromide, and systemic corticosteroid thepary.^4^ In patients who do not respond well to oxygen therapy alone, noninvasive positive pressure ventilation (NPPV) has been effectively used to treat hypoxemia and hypercapnia associated with asthma attacks.^5^, ^6^, ^7^, ^8^, ^9^ Although few reports have described the use of NPPV for acute asthma exacerbations in pregnant women,^10^, ^11^, ^12^ this approach could be beneficial for these patients. Although NPPV is commonly used in clinical practice, definitive evidence regarding its efficacy in pregnant women is lacking. Successful management of asthma exacerbations with NPPV in pregnant women requires careful case selection by experienced clinicians. Here, we report cases of three pregnant women who were hesitant to undergo tracheal intubation because of a fear that their symptoms would be exacerbated by the procedure. In all cases, symptoms of severe dyspnea and hypoxemia caused by acute asthma exacerbation were alleviated with NPPV in a setting in which an expert respiratory nurse and experienced obstetrician and pulmonologist were available.

## 2 CASE EXAMINATION

### 2.1 Patient 1

**Figure 1:**
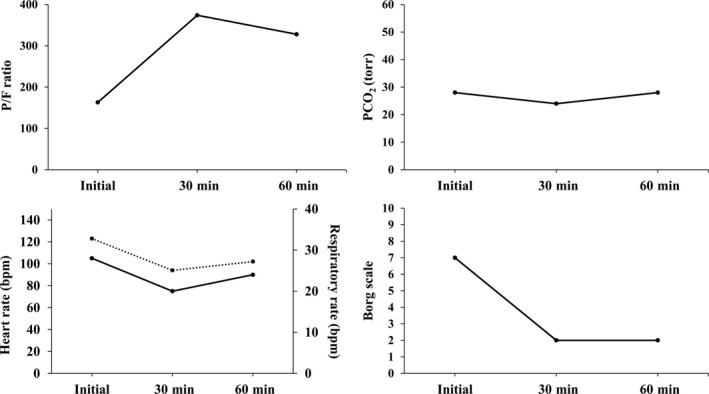
Case 1: Changes in the partial pressure of arterial oxygen/fraction of inspiratory oxygen ratio (P/F ratio), partial pressure of arterial carbon dioxide (PaCO2), respiratory rate, heart rate, and Borg scale level (at the time of initiation and at 30 and 60 min after initiation of NPPV). Lower left panel: solid line demarcates respiratory rate; dotted line demarcates heart rate

Case 1 was a 35‐year‐old (height, 156 cm; weight, 56 kg; BMI, 23 kg/m^2^) pregnant woman. She was 6 months and 26 days pregnant, with no history of smoking or childhood asthma. She lived on an outlying island of Japan close to northwestern Okinawa Main Island. She was diagnosed with bronchial asthma 7 years earlier, at which point treatment with salbutamol and inhaled steroids was initiated. She had recently developed common cold symptoms with yellow sputum. On presentation, she had a 1‐day history of wheezing. She presented to a local clinic, where she received oxygen therapy via mask (5 L/min) and hydrocortisone (100 mg). However, the treatment did not alleviate her symptoms. She subsequently developed low oxygen saturation levels and was transported by helicopter to the emergency department. She was fully conscious and cooperative upon admission to the emergency department. Her main symptoms were dyspnea (Borg scale severity level 7) and tightness in the chest. Her wheezing was categorized as Johnson classification degree II. Her respiratory rate was 28 breaths/min. She was not able to lie down and remained in an orthopneic position. Her body temperature was 36.9°C (98.42°F). Serum C‐reactive protein (CRP) level was 2.43 mg/dL; white blood cell (WBC) count was 20 400 cells/µL. NPPV (V60 Ventilator; Respironics Inc, California, USA) was initiated due to hypoxia (P/F ratio, 163) using an NPPV mask (5 L/min; Confortgel Blue Nasal Mask, Respironics Inc). A nurse with expertise in respiratory care attached NPPV while assessing for the possibility of air leak. NPPV settings used were as follows: S/T mode; inspiratory positive airway pressure (IPAP), 7 cm H_2_O; expiratory positive airway pressure (EPAP), 4 cm H_2_O; inspiratory time (I‐time), 1.0 seconds; and inspired oxygen fraction (FiO_2_), 50%. Hydrocortisone (200 mg) was administered via intravenous drip, and a salbutamol metered‐dose inhaler was incorporated in the NPPV circuit using a respiratory gas mixer (Aero Chamber MV, Trudell Medical International, Canada). Figure 1 shows changes in P/F ratio, PCO_2_, respiratory rate, heart rate, and Borg scale levels at 30 and 60 minutes after the initiation of NPPV therapy. Her P/F ratio dramatically improved during the first 30 minutes along with improvements in PCO_2_, respiratory rate, heart rate, and Borg scale classification. After the initiation of NPPV therapy, her wheezing was resolved at auscultation. He subsequently shifted from the orthopneic to Fowler's position. Fetal heart rate (FHR), as assessed by the obstetrician using Doppler echocardiography, was 152 beats/min. She was transferred from the intensive care unit (ICU) to the general ward. Oxygenation was stabilized to a level that can be coped with low‐flow oxygen therapy, even if NPPV is temporarily discontinued after the P/F ratio is improved. Moreover, re‐exacerbation of wheezing, dyspnea, tachypnea, and vital signs was not observed even if the NPPV mask was removed. Finally, NPPV and oxygen therapies were withdrawn. Bronchitis was suspected to have triggered the acute asthma attack, and she was treated with antibiotics. Inhaled steroids were administered. She was discharged on day 4 post admission. After discharge, her asthma was well controlled, and she was followed up by the doctor at the original clinic, under the advice of a respiratory specialist.

Finally, NPPV and oxygen therapy were withdrawn. Bronchitis was suspected to have triggered the acute asthma attack, and the patient was treated with antibiotics. Inhaled steroids were administered. She was discharged on day 4 post admission. After discharge, her asthma was well controlled, and her follow‐up was conducted by the doctor at the original clinic, under the advice of a respiratory specialist.

### 2.2 Patient 2

Case 2 was a 29‐year‐old (height, 149 cm; weight, 53 kg; BMI, 24 kg/m^2^) pregnant woman. She was 7 months and 2 days pregnant, with a breech presentation. She was a never smoker, with a history of childhood asthma. Pulmonary spirometry revealed the following: forced vital capacity (FVC), 2.5 L; forced expiratory volume % in 1 seconds (FEV1), 1.65 L; and FEV1/FVC ratio, 0.66. She was previously prescribed inhalation therapy with fluticasone; however, she discontinued treatment on her own. She complained of fatigue during childcare over the preceding week. On presentation, she developed symptoms of wheezing and dyspnea at approximately noon. She was subsequently transported to the hospital by ambulance.

On physical examination, she was fully conscious and cooperative. Her main symptoms were dyspnea (Borg scale severity level 9) and wheezing (Johnson classification degree III); her respiratory rate was 36 breaths/min. She was in an anteflexion position. Body temperature was 36.9°C (98.42°F). Serum CRP level was 0.72 mg/dL; WBC count was 122 00 cells/μL. Further deterioration of symptoms compelled the medical team to consider tracheal intubation. NPPV was initiated using a reservoir oxygen mask (10 L/min) to address the patient's hypoxia (P/F ratio, 141). NPPV settings used were as follows: S/T mode; IPAP, 8 cm H_2_O; EPAP, 4 cm H_2_O; I‐time, 0.8 seconds; and FiO_2_, 60%. A nurse with expertise in respiratory care attached the NPPV mask and made the necessary adjustments to prevent air leak and patient discomfort. Methylprednisolone (40 mg) was administered via intravenous drip. Magnesium (20 mEq) was injected intravenously. A hypodermic injection of adrenaline (0.3 mg) was also administered.

**Figure 2:**
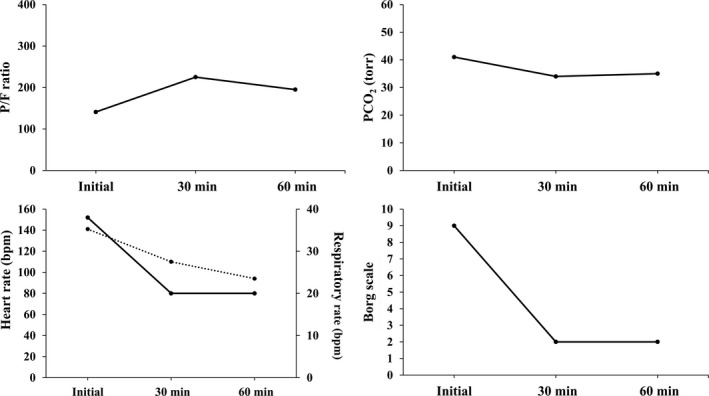
Case 2: Changes in partial pressure of arterial oxygen/fraction of inspiratory oxygen ratio (P/F ratio), partial pressure of arterial carbon dioxide (PaCO2), respiratory rate, heart rate, and Borg scale level (at the time of initiation and at 30 and 60 min after initiation of NPPV). Lower left panel: solid line shows respiratory rate; dotted line shows heart rate

Figure 2 presents changes in P/F ratio, PCO_2_, respiratory rate, heart rate, and Borg scale classification at 30 and 60 minutes after the initiation of NPPV therapy. Borg scale classification and respiratory rate dramatically improved over the first 30 minutes along with improvements in P/F ratio, PCO_2_, and heart rate. Under NPPV therapy, her wheezing improved to Johnson classification degree II at auscultation, and she shifted from the anteflexion to Fowler's position. FHR, as assessed by an obstetrician using Doppler echocardiography, was 146 beats/min. She was subsequently transferred from ICU to the obstetric ward. Oxygenation was stabilized to a level that can be coped with low‐flow oxygen therapy, even if NPPV is temporarily discontinued after the P/F ratio is improved. Moreover, re‐exacerbation of wheezing, dyspnea, tachypnea, and vital signs was not observed even if the NPPV mask was removed. NPPV therapy was discontinued, and oxygen therapy was initiated. She was treated with sultamicillin tosilate hydrate (1500 mg) for a suspected infection. Ultimately, oxygen therapy was also withdrawn, and she maintained percutaneous oxygen saturation (SpO_2_) of 97% on room air. She was discharged on day 5 post admission with a prescription for prednisolone (30 mg) and a budesonide dry‐powder inhaler. She safely delivered a female child weighing 2490 g (Apgar score 9/9) at gestational age of 9 months and 1 day via cesarean section performed under lumbar anesthesia. After discharge, she was treated as an outpatient by a respiratory specialist. She has continued to adhere to the prescribed regimen, and her asthma remains well controlled.

### 2.3 Patient 3

Case 3 was a 20‐year‐old (height, 158 cm; weight, 51 kg; BMI, 21 kg/m^2^) pregnant female. She was 9 months and 2 days pregnant. She was a never smoker, with a history of childhood asthma and allergic rhinitis. Pulmonary spirometry revealed the following: FVC, 2.45 L; FEV1, 0.96 L; and FEV1/FVC ratio, 0.39. She was treated for asthma until 17 years of age; however, the treatment was then interrupted. She had developed influenza 2 weeks before presentation. She complained of nocturnal cough, which eventually resolved. On the day of presentation, she suddenly developed dyspnea around noon and was transported to the hospital by ambulance.

**Figure 3:**
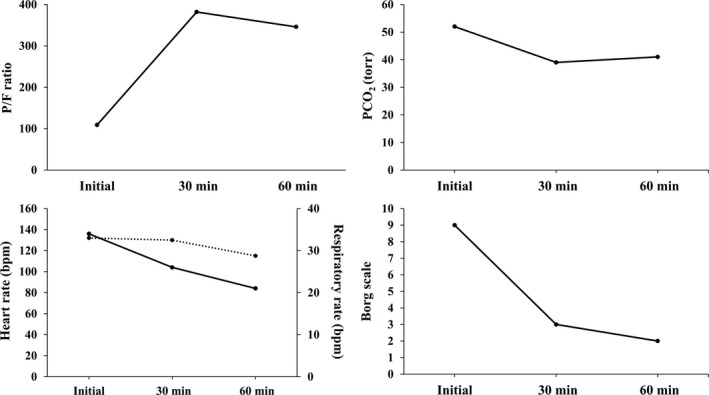
Case 3: Changes in partial pressure of arterial oxygen/fraction of inspiratory oxygen ratio (P/F ratio), partial pressure of arterial carbon dioxide (PaCO2), respiratory rate, heart rate, and Borg scale classification (at the time of initiation and at 30 and 60 min after initiation of NPPV). Lower left panel: solid line shows respiratory rate; dotted line shows heart rate

She was fully conscious at the time of admission to the emergency department. Her main symptoms were dyspnea (Borg scale severity level 9) and wheezing (Johnson classification degree III); her respiratory rate was 34 breaths/min. Her neck accessory muscles (scalene and sternocleidomastoid) were prominently contracted. She was in an anteflexion position. Her body temperature was 37°C (98.6°F). Serum CRP level was 0.45 mg/dL; WBC count was 7700 cells/μL. Arterial blood gas analysis during reservoir oxygen mask therapy (10 L/min) revealed acute decompensated respiratory acidosis with hypercapnia (pH, 7.28; PaO_2_, 109 mm Hg; sat, 97%; PaCO_2_, 52 mm Hg; and HCO_3_−, 24.4). Her P/F ratio was 109. NPPV was initiated via face mask to address her low P/F ratio and hypercapnia. The nurse attached the NPPV mask, while evaluating it for air leak. The NPPV settings were as follows: S/T mode; IPAP, 10 cm H_2_O; EPAP, 4 cm H_2_O; I‐time, 0.8 seconds; and FiO_2_, 100%. Methylprednisolone (40 mg) was administered via intravenous drip, and adrenaline (0.3 mg) was administered via hypodermic injection. Figure 3 presents changes in P/F ratio, PCO_2_, respiratory rate, heart rate, and Borg scale level, at 30 and 60 minutes after the initiation of NPPV therapy. P/F ratio (382), PCO_2_ (39 mm Hg), and Borg scale level (grade 2) dramatically improved within the first 30 minutes along with improvements in respiratory and heart rates over time. Under NPPV therapy, her wheezing improved to Johnson classification degree II. The patient's position shifted from the anteflexion to Fowler's position. Prominent contraction of accessory respiratory muscles resolved. FHR, as estimated by an obstetrician using Doppler echocardiography, was 150 beats/min. The patient was transferred from the ICU to the obstetric ward. Oxygenation was stabilized to a level that can be coped with low‐flow oxygen therapy, even if NPPV is temporarily discontinued after the P/F ratio is improved. Moreover, re‐exacerbation of wheezing, dyspnea, tachypnea, and vital signs was not observed even if the NPPV mask was removed. Additionally, hypercapnia and respiratory acidosis were not re‐exacerbated after NPPV is discontinued temporarily. NPPV therapy was discontinued and replaced with oxygen therapy. Ultimately, oxygen therapy was also withdrawn. The patient was discharged on day 8 post admission and prescribed budesonide and a formoterol fumarate hydrate dry‐powder inhaler. The patient safely delivered a female child weighing 3006 g (Apgar score 8/9) at gestational age of 10 months and 5 days via spontaneous cephalic delivery. After discharge, the patient's condition was well maintained under the care of a respiratory specialist. The patient has adhered to the prescribed regimen of asthma medication.

## 3 DISCUSSION

We present three cases of pregnant women in whom symptoms of severe dyspnea and hypoxemia due to acute asthma exacerbation were alleviated following NPPV in addition to standard therapy. Although NPPV is a ventilatory support that is being increasingly used during acute asthma attacks,^5^, ^6^, ^7^, ^8^, ^9^ a definitive consensus is lacking in guidelines.^10^ This is because, regarding the results of several previous reports, NPPV for asthma attacks are observational research and cohort research subjects, and only a small number of randomized controlled trials (RCTs) are on the scale of 10‐20 patients.^6^, ^11^ Furthermore, we believe that outcomes obtained from RCTs are mostly lung function improvement because clinically significant results such as decreased intubation and mortality rates have not been obtained. However, we believe that the effect of NPPV on asthma attacks cannot be completely denied. As clinical cases, a few case reports have described the use of NPPV for management of acute exacerbations related to asthma in pregnant women.^12^, ^13^, ^14^ Moreover, we believe that hospital staff members are not necessarily proficient in administering NPPV. Therefore, it cannot be generally recommended to all pregnant women with asthma exacerbations, and careful case selection is therefore important. We have to keep in mind that NPPV is a supportive therapy that is used together with conventional pharmacological treatment, aiming at decreasing the respiratory muscle work that is much increased during the episodes of acute bronchoconstriction, to improve ventilation and decrease the sensation of dyspnea, and it may be able to avoid intubation and invasive mechanical ventilation. The ideal patient is the one who is fully conscious and cooperative. The facility should have an appropriate system for monitoring vital signs and preparation for emergency intubation and invasive mechanical ventilation within a critical care environment. Furthermore, the availability of expert respiratory care nurses capable of administering NPPV is crucial to ensure the success of NPPV in pregnant women with asthma exacerbations. Furthermore, the presence of experienced obstetrician and pulmonologist are recommended under such circumstances.^15^ In the first two of our three cases, the patients suffered from type I respiratory failure. A recent report had used high‐flow oxygen therapy for asthma.^16^ However, we attempted to use NPPV for asthmatic attacks because we expected the following four effects: (a) Applying positive pressure (Expiratory Positive Airway Pressure [EPAP]) in the trachea may cause bronchodilation and reduce airway resistance^17^; (b) β‐2‐agonists use by incorporating them in the NPPV circuit using a respiratory gas mixer^18^; (c) For the endogenous positive end‐expiratory pressure (endogenous PEEP) during an asthmatic attack, EPAP acts to counter endogenous PEEP to improve wheezing and labored exhalation; and (d) Respiratory muscle fatigued with tachypnea is assisted by inspiratory positive airway pressure (IPAP).^19^ All three cases described in this report presented at the emergency department with hypoxemia and severe dyspnea under oxygen therapy. Nevertheless, all were fully conscious and cooperative. FHR was closely monitored by an experienced obstetrician using Doppler echocardiography.^15^ Pregnant women and their spouses were informed of their physical status by the pulmonologist. Treatment with appropriate standard medications combined with NPPV therapy was initiated to alleviate hypoxemia and strong dyspnea. Patients were also informed about the potential need for intubation and invasive mechanical ventilation in case of respiratory failure or fatigue. Intubation and invasive mechanical ventilation are effective treatments for respiratory failure. However, intubation is risky in pregnant women due to pregnancy‐related airway hyperemia and aspiration during intubation. Moreover, the clinician should be aware that hypotension may follow positive pressure ventilation and sedation. Some sedatives are not safe for use during pregnancy.^20^ The use of intubation and invasive mechanical ventilation was avoided in these cases in part because of the early initiation of NPPV (within 30 minutes). This approach led to dramatic improvements in parameters, such as oxygenation, hypercapnia, respiratory rate, heart rate, and dyspnea index. NPPV combined with asthma and medication therapies may become an effective treatment for asthma attacks in pregnant women although careful case selection is imperative. This report has certain limitations. We studied only three cases. Moreover, we were fortunate to achieve good clinical outcomes with all three patients. We emphasize that NPPV cannot be universally recommended for all pregnant women who present with acute asthma exacerbations. Moreover, NPPV via mask, which does not separate the trachea and esophagus, always carries a risk of aspiration while treating pregnant women. A key factor in all these cases was the availability of expert respiratory care nurses who were familiar with the application of NPPV masks as well as operation of necessary equipment. Moreover, the patients had access to a pulmonologist specializing in asthma treatment and management of respiratory failure.

This study investigated the utility of NPPV therapy in addition to standard pharmacologic therapy for acute asthma exacerbations in pregnant women with dyspnea and hypoxemia compared with oxygen therapy alone. Careful patient selection and clinicians’ NPPV experience are crucial for optimizing patient outcomes.

## CONFLICT OF INTEREST

None declared.

## AUTHOR CONTRIBUTION

HS: drafted the manuscript. MB, YS, and TM: treated the patient. KH: analyzed and interpreted the data; YK, TF, and IK: made critical revisions of the manuscript.

## ETHICAL STATEMENT

Our case reports were approved by the Ethics Committee of Tomishiro Central Hospital (number H29R026) and were performed according to the ethical standards of the Declaration of Helsinki. All patients provided written informed consent for the publication of this case report and its accompanying images.
